# Long non-coding RNA LINC01419 mediates miR-519a-3p/PDRG1 axis to promote cell progression in osteosarcoma

**DOI:** 10.1186/s12935-020-01203-0

**Published:** 2020-05-05

**Authors:** Zhiqian Gu, Shaokun Wu, Jingnan Wang, Shoujun Zhao

**Affiliations:** 1 Department of Orthopedics, Hwa Mei Hospital, University of Chinese Academy of Sciences (Ningbo NO. 2 Hospital), No. 41 Northwest Street, Ningbo, 315010 Zhejiang China; 2 Ningbo Institute of Life and Health Industry, No. 41 Northwest Street, Ningbo, 315010 Zhejiang China

**Keywords:** LINC01419, miR-519a-3p, PDRG1, Osteosarcoma

## Abstract

**Background:**

Osteosarcoma (OS) is one of the most aggressive malignancies with mortality rate worldwide. Accumulating evidence has revealed that long noncoding RNAs (lncRNAs) exert important functions in regulation of cancer initiation and progression. Recently, long intergenic non-protein coding RNA 1419 (LINC01419) has been reported to function as an oncogene in several cancers. However, its role in OS has not been explored yet.

**Methods:**

qRT-PCR and western blot analyses were implemented to determine the expression of genes. The function of OS cells was assessed through colony formation, EdU, JC-1, TUNEL, transwell, and immunofluorescence (IF) assays. FISH and subcellular fractionation assays were conducted to estimate the localization of LINC01419 in OS cells. The interaction between genes was validated through luciferase reporter and RNA pull down assays.

**Results:**

LINC01419 expression was elevated in OS tissues and cells. Functionally, LINC01419 accelerated OS cell proliferation, motility and EMT. In vivo assay showed that silencing LINC01419 hindered the growth of OS tumors. Mechanistic investigation unveiled that LINC01419 acted as a competing endogenous RNA (ceRNA) to augment PDRG1 expression by miR-519a-3p sequestration. Rescue assays verified the oncogenic effect of LINC01419/miR-519a-3p/PDRG1 axis on OS development.

**Conclusion:**

LINC01419 mediates malignant phenotypes in OS by targeting miR-519a-3p/PDRG1 axis.

## Background

Osteosarcoma (OS), a commonly diagnosed malignancy in skeletal system, mainly occurs in adolescents or children and affects the bone growth [1]. In pediatric bone malignancies, OS occupies about 5% of pediatric cancers [1]. In recent years, the 5-year survival rate of OS patients has been continuously improved because of the introduction of resection surgery and chemoradiotherapy [2]. Nevertheless, obtained chemoresistance and high recurrence rate caused by distant metastasis significantly reduced the survival rate of most OS patients [3, 4]. Therefore, it is urgent for us to explore new molecular mechanisms underlying the pathogenesis and progression of OS.

Recent studies have demonstrated that over 98% of human transcriptome lacking protein-coding ability are recognized as non-coding RNAs (ncRNAs) [5]. In addition, ncRNAs comprise long non-coding RNAs (lncRNAs) and microRNAs (miRNAs) [6–8]. LncRNAs are a group of ncRNAs with more than 200 nucleotides in length [9]. Accumulating studies demonstrated that lncRNAs could function in many cancers to affect the progression of cancers, such as epithelial ovarian cancer, papillary thyroid cancer and gastric cancer [10–12]. It has been proved that lncRNAs that are aberrantly regulated in cancer could modulate diverse biological behaviors, such as cell proliferation, apoptosis, migration and epithelial-mesenchymal transition (EMT) [13, 14]. In mechanisms, lncRNAs could regulate gene expression through gene imprinting or epigenetic modification. Besides, increasing studies proposed that lncRNA could function as a competing endogenous RNA (ceRNA) by sponging miRNAs to elevate target gene expression. For instance, lncRNA PVT1 sponged miR-424-5p to up-regulate CARM1 and then modulate the development of non-small-cell lung cancer [15]. LncRNA SNHG5 regulated miR-26a-5p/TRPC3 axis to facilitate cell growth and invasion in melanoma [16]. Long intergenic non-protein coding RNA 1419 (LINC01419) has been demonstrated as an oncogene in lung adenocarcinoma, esophageal squamous cell carcinoma and gastric cancer [17–19]. However, its role and possible molecular mechanism have not been studied in OS yet.

In this study, we probed into the function of LINC01419 in OS and found the high expression trend and tumor-facilitating role of LINC01419 in OS, providing a new thought for future OS treatment.

## Methods

### Tissue samples

The OS tissues (n = 50) and adjacent non-tumor tissues (n = 50) were collected from September 2012 to August 2017 in the Hwa Mei Hospital, University of Chinese Academy of Sciences (Ningbo NO. 2 Hospital). All patients accepted no any therapy before surgery and have signed informed consent prior to this study. Samples were sharply frozen in liquid nitrogen and then preserved in − 80 °C. This study was permitted and approved by the Ethics Committee of Hwa Mei Hospital, University of Chinese Academy of Sciences (Ningbo NO. 2 Hospital).

### Cell lines

Human OS cell lines (143B, U2OS, Saos-2) and normal osteoblast (hFOB1.19) were commercially acquired from ATCC (Rockville, Maryland). Saos-2 was maintained in DMEM (Invitrogen, Carlsbad, CA) with 15% FBS (Gibco, Grand Island, NY) and 1% antibiotics, whereas other cells were grown in DMEM (Invitrogen) with 10% FBS (Gibco) and 1% antibiotics. All cells were incubated under 5% CO_2_ at 37 °C.

### RNA extraction and qRT-PCR

The total RNAs were extracted from OS cells, tissue samples or in vivo xenografts in line with the manual of TRIzol reagent (Thermo Fisher Scientific, Waltham, MA), for converting into cDNA. qRT-PCR was implemented using Power SYBR Green (TaKaRa, Shiga, Japan) on the Step-One Plus Real-Time PCR System (Applied Biosystems, Foster City, CA). RNA expressions were calculated by 2^−ΔΔCt^ method, and U6/GAPDH was the internal control. The primers were presented in Table 1.

**Table 1:** Sequences of primers used in qRT-PCR and of plasmids applied in cell transfection

Genes	Sequences of primers (5′–3′)
LINC01419	F: AGAACTGAGGTCCACTTTCTGG
	R: GGTCCTTTGTCTGCAACGA
miR-519a-3p	F: AAAGTGCATCCTTTTAGAGTGTGG
	R: CTCTACAGCTATATTGCCAGCCAC
miR-519c-3p	F: AAAGTGCATCTTTTTAGAGGATCG
	R: CTCTACAGCTATATTGCCAGCCAC
miR-519b-3p	F: AAAGTGCATCCTTTTAGAGGTTCC
	R: CTCTACAGCTATATTGCCAGCCAC
miR-3121-3p	F: TAAATAGAGTAGGCAAAGGACAGCC
	R: CTCTACAGCTATATTGCCAGCCAC
PDRG1	F: TGAGCCACAAAGCACCAA
	R: TCACATGGACCCTGAGCA
GAPDH	F: CATCCTGGGCTACACTGAGC
	R: AGTGGTCGTTGAGGGCAA
U6	F: AAAGCAAATCATCGGACGACC
	R: GTACAACACATTGTTTCCTCGGA

shRNAs/pcDNA3.1	Sequences (5′-3′)
sh-NC	CCGGCACTAATGTCTGCGTCAATGTCTCGAGACATTGACGCAGACATTAGTGTTTTTG
sh-LINC01419#1	CCGGTGCATAGATTCATTAAGAATTCTCGAGAATTCTTAATGAATCTATGCATTTTTG
sh-LINC01419#2	CCGGATCTATGAAGCCAAACCTAATCTCGAGATTAGGTTTGGCTTCATAGATTTTTTG
sh-NC	CCGGGTGACATAGTGCACAGTTGGACTCGAGTCCAACTGTGCACTATGTCACTTTTTG
sh-PDRG1#1	CCGGCACCAAGATTCTGTTCTTCATCTCGAGATGAAGAACAGAATCTTGGTGTTTTTG
sh-PDRG1#2	CCGGACCAAGATTCTGTTCTTCATTCTCGAGAATGAAGAACAGAATCTTGGTTTTTTG
pcDNA3.1-P DRG1	ATGCTATCACCCGAGGCAGAGCGAGTGCTGCGGTACCTTGTAGAAGTGGAGGAGCTCGCCGAGGAGGTGCTGGCGGACAAGCGGCAGATTGTGGACCTGGACACTAAAAGGAATCAGAATCGAGAGGGCCTGAGGGCCCTGCAGAAGGATCTCAGCCTCTCTGAAGATGTGATGGTTTGCTTCGGGAACATGTTTATCAAGATGCCTCACCCTGAGACAAAGGAAATGATTGAAAAAGATCAAGATCATCTGGATAAAGAAATAGAAAAACTGCGGAAGCAACTTAAAGTGAAGGTCAACCGCCTTTTTGAGGCCCAAGGCAAACCGGAGCTGAAGGGTTTTAACTTGAACCCCCTCAACCAGGATGAGCTTAAAGCTCTCAAGGTCATCTTGAAAGGA
Mimics/inhibitor	
NC mimics	AUUCACGUUCCUUUUAGAGUGU
miR-519a-3p mimics	AAAGUGCAUCCUUUUAGAGUGU
NC inhibitor	ACACUCUAAAAGGAACGUGAAU
miR-519a-3p inhibitor	ACACUCUAAAAGGAUGCACUUU

### Transfection

The specific shRNAs against LINC01419 (sh-LINC01419#1/2) or PDRG1 (sh-PDRG1#1/2) and pcDNA3.1-PDRG1, as well as their negative control (NC) including the nonspecific shRNA (sh-NC) and empty pcDNA3.1 vector, were all procured from RiboBio (Guangzhou, China). The miR-519a-3p mimics/inhibitor and NC mimics/inhibitor were acquired from Genepharma (Shanghai, China). Thereafter, above plasmids were transfected into the U2OS and Saos-2 cells for 48 h by use of Lipofectamine 2000 (Invitrogen, Carlsbad, CA), as appropriate. The sequences were shown in Table 1.

### Colony formation assay

Cells were seeded in the 6-well culture plates (1 × 10^3^ cells/well) and incubated for 2 weeks. Afterwards, colonies were colored via crystal violet solution after being fixated by 4% paraformaldehyde. Finally, colonies with over 50 cells were manually calculated.

### EdU assay

Cells were plated into the 96-well culture plates and added with the EdU solution (Ribobio) for 3 h and then treated with 4% paraformaldehyde. Following permeabilization, cells were incubated with the DAPI solution and then analyzed by fluorescent microscope (Leica, Wetzlar, Germany).

### JC-1 assay

Transfected cells were harvested for culturing with the 10 nM of JC-1 solution (Beyotime, Shanghai, China). Following 30 min of incubation at 37 °C, samples were tagged with fluorescence and rinsed in PBS for EnSpire Reader analysis (PerkinElmer, Waltham, MA).

### TUNEL assay

According to the instruction of in situ cell death detection kit (Roche, Basel, Switzerland), cells were fixed for 1 h, permeabilized for 2 min, and then cultivated with TUNEL assay buffer for 1 h. Following DAPI staining, apoptotic cells were monitored under fluorescent microscope (Leica).

### Transwell assays

Transfected cells were seeded into the top transwell inserts (Corning Incorporated, Corning, NY) with Matrigel membrane (BD Biosciences, Franklin Lakes, NJ) for invasion assay, or without Matrigel for migration assay. The complete medium was supplemented to the bottom inserts. 24 h post-incubation, migrated or invaded cells were treated with 4% paraformaldehyde and crystal violet for observation under microscope.

### Immunofluorescence (IF)

Cells were seeded in the 12-well culture plates, and then processed with 4% formaldehyde in PBS, with 0.5% Triton X-100 for 10 min. Following being mixed with 5% bovine serum albumin (BSA) for 30 min, the specific antibodies to E-cadherin (ab76055, 1:500 dilution; Abcam, Cambridge, UK) and N-cadherin (ab76057, 1:500 dilution; Abcam), as well as the secondary antibodies (ab205719, 1:2000 dilution; Abcam) were used in sequence. Samples treated with DAPI solution were analyzed under microscope.

### Western blot

Total proteins were extracted from U2OS and Saos-2 cells by RIPA buffer. Then, extracted proteins were separated using SDS-PAGE and transferred onto PVDF membranes (Millipore, Billerica, MA, USA). After being sealed via 5% skim milk, the membranes were processed with primary antibodies (Abcam, Cambridge, USA) overnight at 4 °C. Later, the membranes were added with secondary antibodies (ab205719, 1:10000; Abcam) at 37 °C in a dark room for 1 h of incubation. At last, the protein bands were visualized by enhanced ECL system (Tanon, Shanghai, China). The internal control was GAPDH. Primary antibodies were as below: E-cadherin (ab76055, 1:1000 dilution), N-cadherin (ab76057, 1:1000 dilution), Vimentin (ab92547, 1:2000 dilution), β-catenin (ab16051, 1:2000 dilution), GAPDH (ab181602, 1:10000 dilution). All these antibodies were all purchased from Abcam.

### In vivo assay

Ten BALA/C nude male mice (aged 4-6 weeks; weighted 18–24 g) were acquired from Shi Laike Company (Shanghi, China). Then, the mice were randomly divided into 2 groups (n = 5 per group) and injected subcutaneously with U2OS cells which were stably transfected with sh-LINC01419 or sh-NC. Every 4 days, tumor size was monitored and recorded. 28 days later, the mice were sacrificed through cervical dislocation and the tumor was excised and weighed. Tumor volume was assessed according to the formula (mm^3^): tumor volume = length × width^2^ × 0.5. The animal experiments were approved by Hwa Mei Hospital, University of Chinese Academy of Sciences (Ningbo NO.2 Hospital).

### Immunohistochemistry (IHC)

IHC was conducted as described previously [20]. Briefly, sections from paraffin-embedded xenografts were processed with primary antibodies against Ki-67 (ab15580, 1:500 dilution; Abcam) and PCNA (ab92552, 1:500 dilution; Abcam). The proteins in situ were observed with the application of Super Sensitive Link-Label IHC Detection System (BioGenex, Fremont, CA, USA).

### FISH

The FISH probe designed for LINC01419 was commercially obtained from Ribobio and used as per the user guide. DAPI solution was added for nuclear counterstaining. Images were captured under microscope.

### Subcellular fractionation

Based on the supplier’s instruction, PARIS™ Kit (Invitrogen) was employed for subcellular fractionation assay. The distribution of RNAs in nuclei and cytoplasm was monitored by qRT-PCR.

### MS2-RIP assay

Cells were transfected, and then harvested for the co-transfection with pcDNA3.1-MS2 and pcDNA3.1-MS2-LINC01419 for 48 h. Samples were then subjected to RIP assay with GFP antibody (ab290, 1:200 dilution; Abcam) by use of RIP RNA-Binding Protein Immunoprecipitation Kit (Millipore, Bedford, MA). Finally, qRT-PCR was exploited to analyze the level of co-precipitated RNAs.

### RNA pull down assay

The wild-type (WT) and mutated (Mut) miR-519a-3p fragments covering the LINC01419 or PDRG1 were synthesized and biotinylated to Bio-miR-519a-3p-WT/Mut. The probes were cultivated with the protein extracts from U2OS and Saos-2 cells, followed by incubation with the streptavidin agarose magnetic beads for 1 h. RNA enrichment in pull-downs was detected via qRT-PCR.

### Dual-luciferase reporter gene analyses

The recombinant luciferase reporter vectors LINC01419-WT/Mut and PDRG1-WT/Mut were severally established by using the wild-type and mutated LINC01419 or PDRG1 fragments covering miR-519a-3p binding sites. The Dual-Luciferase miRNA Target Expression Vector pmirGLO was obtained from Promega (Madison, WI). The reporter vectors were co-transfected with miR-519a-3p mimics or NC mimics for 48 h, and then luciferase activity of the reporters was monitored via Dual-Luciferase Reporter Assay System (Promega), respectively.

### Statistical analyses

Data from three independent bio-triplications were exhibited as the mean ± standard deviation (SD). Statistical difference was analyzed by Student’s *t* test or one-way ANOVA via PRISM 6 (GraphPad, San Diego, CA), with the p < 0.05 meant statistically significant.

## Results

### LINC01419 acts as an oncogene in OS

**Fig. 1:**
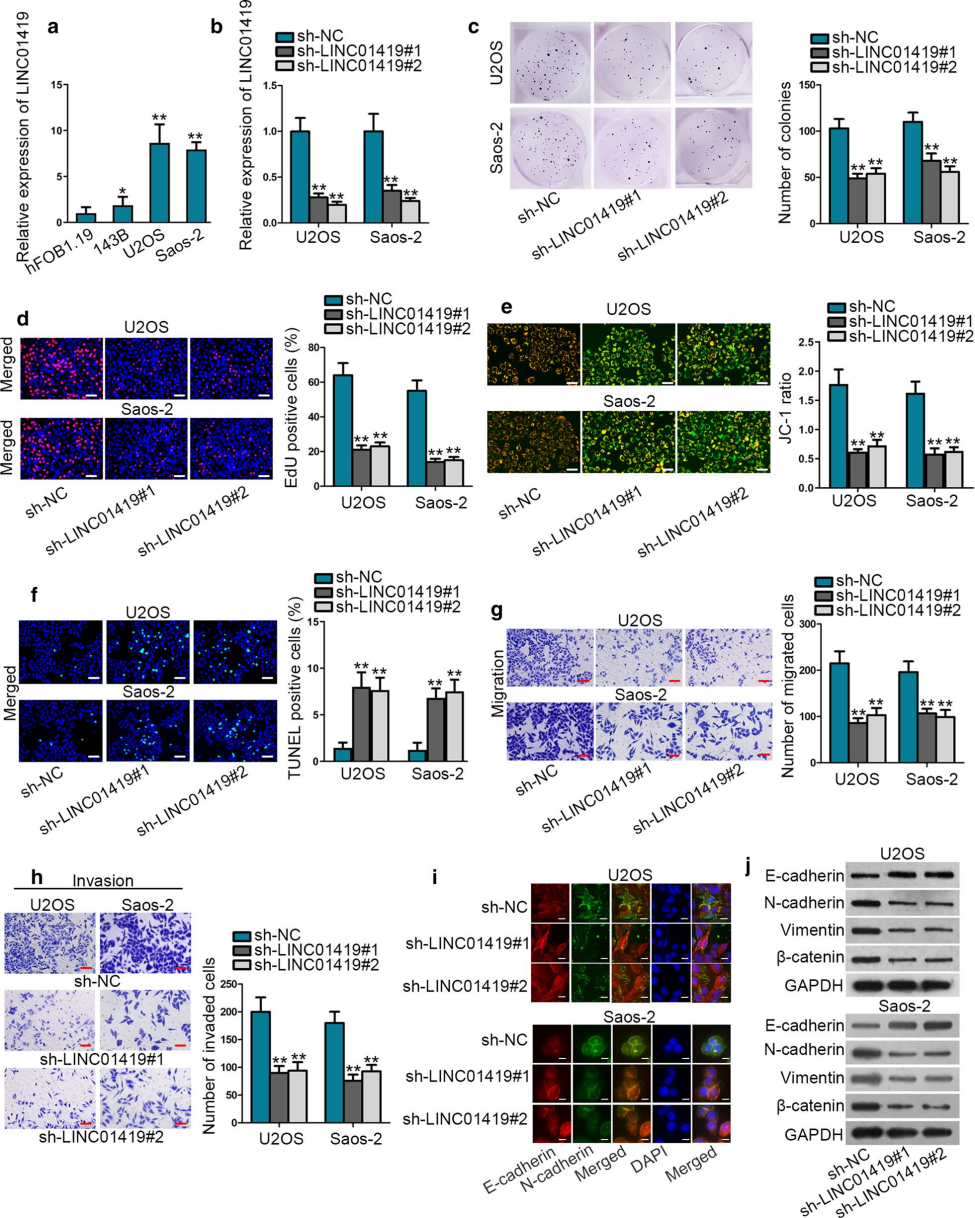
LINC01419 acts as an oncogene in OS. **a** LINC01419 expression was assessed in OS cell lines (143B, U2OS and Saos-2) and human normal osteoblast (hFOB1.19) by using qRT-PCR analysis. **b** Silencing efficiency of LINC01419 was evaluated in U2OS and Saos-2 cells by qRT-PCR. **c–d**. Colony formation assay and EdU assay (scale bar = 200 μm) were conducted to evaluate the proliferation of U2OS and Saos-2 cells transfected with sh-LINC01419#1/2 or sh-NC. **e**–**f**. U2OS and Saos-2 cell apoptosis was examined by JC-1 assay (scale bar = 200 μm) and TUNEL assay (scale bar = 200 μm) in sh-LINC01419 group or sh-NC group. **g**–**h**. U2OS and Saos-2 cells migration and invasion were illustrated by transwell assay (scale bar = 200 μm) with the transfection sh-LINC01419 or sh-NC. **i–j**. IF (scale bar = 50 μm) and western blot assay were conducted to investigate the EMT process of sh-LINC01419 or sh-NC transfected U2OS and Saos-2 cells. ^*^P < 0.05, ^**^P < 0.01

Firstly, we determined the expression pattern of LINC01419 in OS. It was showed that LINC01419 exhibited a higher expression level in OS tissues than in adjacent non-tumor tissues (Additional file 1: Figure S1A). Then, we checked LINC01419 expression in OS cell lines. As a result, LINC01419 was significantly up-regulated in OS cell lines relative to normal hFOB1.19 cells, especially in U2OS and Saos-2 cells (Fig. 1a). Based on above results, we wanted to further explore the biological role of LINC01419 in OS. Prior to that, we confirmed that LINC01419 was successfully silenced in U2OS and Saos-2 cells after transfection with sh-LINC01419#1/2 (Fig. 1b). Then, the proliferation of U2OS and Saos-2 cells was determined by colony formation assay and EdU assay. The results manifested that LINC01419 suppression led to inhibited OS cell proliferation (Fig. 1c, d). By contrast, JC-1 and TUNEL assays revealed that the apoptosis of U2OS and Saos-2 cells was hastened upon LINC01419 knockdown (Fig. 1e, f). Moreover, transwell assay delineated that the migration and invasion were curbed in U2OS and Saos-2 cells after silencing LINC01419 (Fig. 1g, h). Through IF assay, we observed that EMT process was hampered by silenced LINC01419 (Fig. 1i). Furthermore, western blot analysis indicated that LINC01419 deficiency augmented the protein level of E-cadherin but lowered the protein levels of N-cadherin, Vimentin and β-catenin (Fig. 1j). These results suggested that LINC01419 enhanced OS cell malignancies in vitro. Meanwhile, we carried out in vivo assay to further validate the contributing role of LINC01419 in OS. Consequently, tumors excised from mice injected with LINC01419-silenced cells were much smaller than those from control group (Additional file 1: Figure S1B). Also, decreased volume and weight was observed in tumors from group with LINC01419 depletion compared to controls (Additional file 1: Figure S1C-D). In addition, it was confirmed that LINC01419 expression in tumors originated from sh-LINC01419#1-transfected cells was much less than in those from control group (Additional file 1: Figure S1E). Moreover, IHC assay implied that LINC01419 silencing reduced the positivity of PCNA and Ki-67 in in vivo tumors (Additional file 1: Figure S1F). Taken all together, LINC01419 served as an oncogene in OS.

### LINC01419 sponges miR-519a-3p in OS

**Fig. 2:**
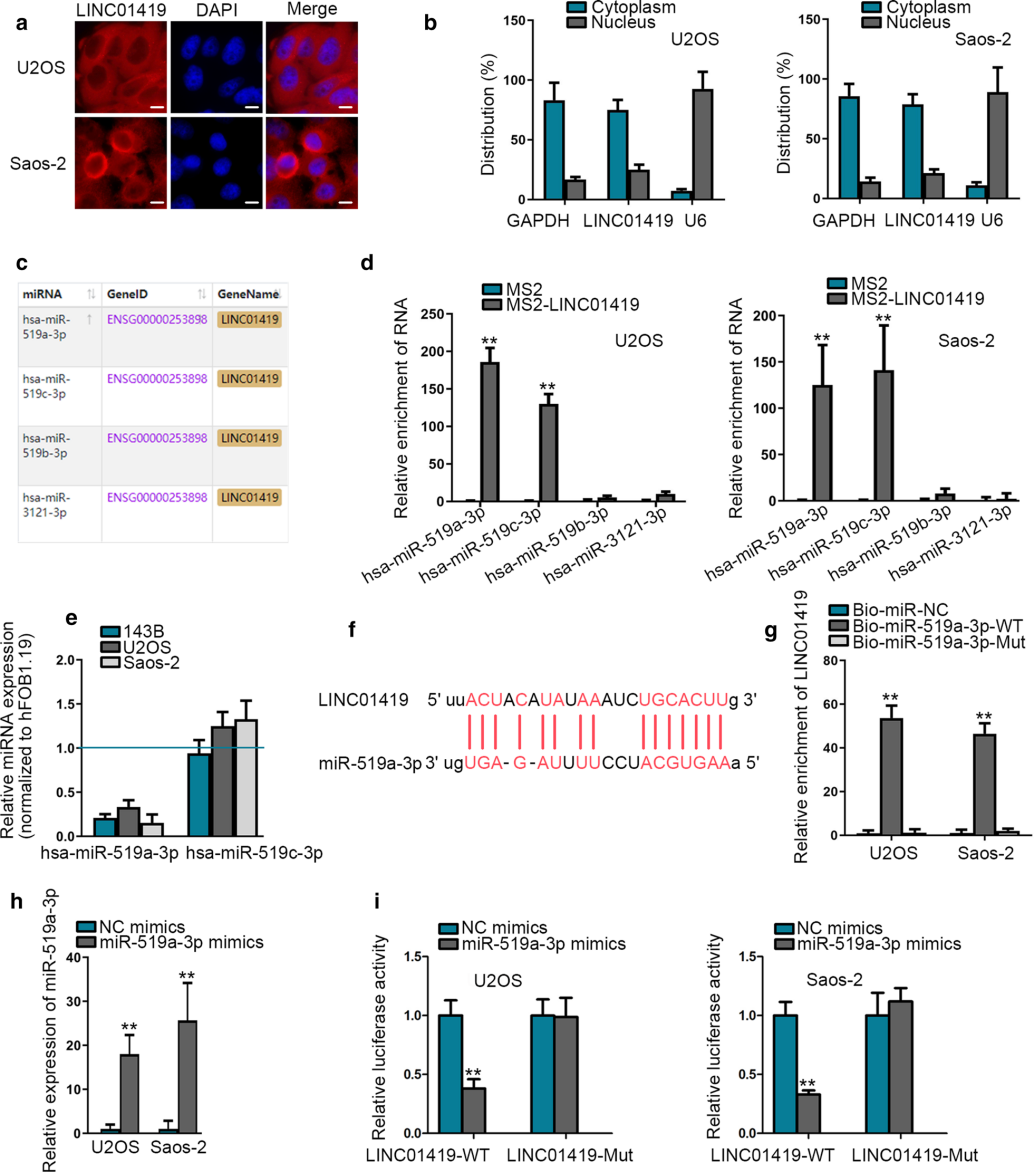
LINC01419 sponges miR-519a-3p in OS. **a**–**b** FISH assay (scale bar = 50 μm) and subcellular fractionation assay were implemented to determine the location of LINC01419 in OS cells. **c** Four miRNAs that bound to LINC01419 were screened out from ENCORI database. **d** MS2-RIP assay was conducted to assess the binding of 4 mirnas to linc01419. **e** Expressions of miR-519a-3p and miR-519c-3p in OS cells were investigated via qRT-PCR. **f** Binding sites between LINC01419 and miR-519a-3p were predicted by ENCORI database. **g** RNA pull down assay was implemented to evaluate the interaction between LINC01419 and miR-519a-3p in OS cells. **h** Overexpression efficiency of miR-519a-3p in U2OS and Saos-2 cells was examined by qRT-PCR. **i** Luciferase reporter assay verified the binding of LINC01419 to miR-519a-3p in U2OS and Saos-2 cells. ^**^P < 0.01

Next, we explored the molecular mechanism of LINC01419 in OS. It has been evidenced that lncRNA could serve as a ceRNA to up-regulate mRNA expression via sponging miRNAs in the cytoplasm [21, 22]. First of all, we evaluated the distribution of LINC01419 by FISH and subcellular fractionation analyses. The results disclosed that a larger proportion of LINC01419 was located in the cytoplasm than in nucleus of both the two OS cells (Fig. 2a, b). Thence, we speculated that LINC01419 might act as a miRNA sponge. According to ENCORI database (http://starbase.sysu.edu.cn/index.php), four potential miRNAs (miR-519a-3p, miR-519c-3p, miR-519b-3p and miR-3121-3p) were postulated to bind to LINC01419 (Fig. 2c). To screen out the miRNAs interacted with LINC01419 in OS, MS2-RIP assay was conducted and verified the abundant enrichment of miR-519a-3p and miR-519c-3p in LINC01419-precipitated compounds in these two cells (Fig. 2d). Through qRT-PCR assay, miR-519a-3p was found to be lowly expressed in OS cells while miR-519c-3p showed no expression difference (Fig. 2e). Therefore, miR-519a-3p was chosen for further exploration. As demonstrated in Fig. 2f, binding sites between LINC01419 and miR-519a-3p were predicted via ENCORI database. RNA pull down assay uncovered a direct interaction between miR-519a-3p and LINC01419 whereas mutated miR-519a-3p had no capacity to bind with LINC01419 (Fig. 2g). Moreover, miR-519a-3p overexpression efficiency was validated by qRT-PCR assay (Fig. 2h). Luciferase reporter assay verified that luciferase activity of LINC01419-WT was significantly lessened by ectopic expression of miR-519a-3p whereas no difference was presented in that of LINC01419-Mut (Fig. 2i). In conclusion, LINC01419 could sponge miR-519a-3p in OS.

### PDRG1 is targeted by miR-519a-3p and promotes OS cell progression

**Fig. 3:**
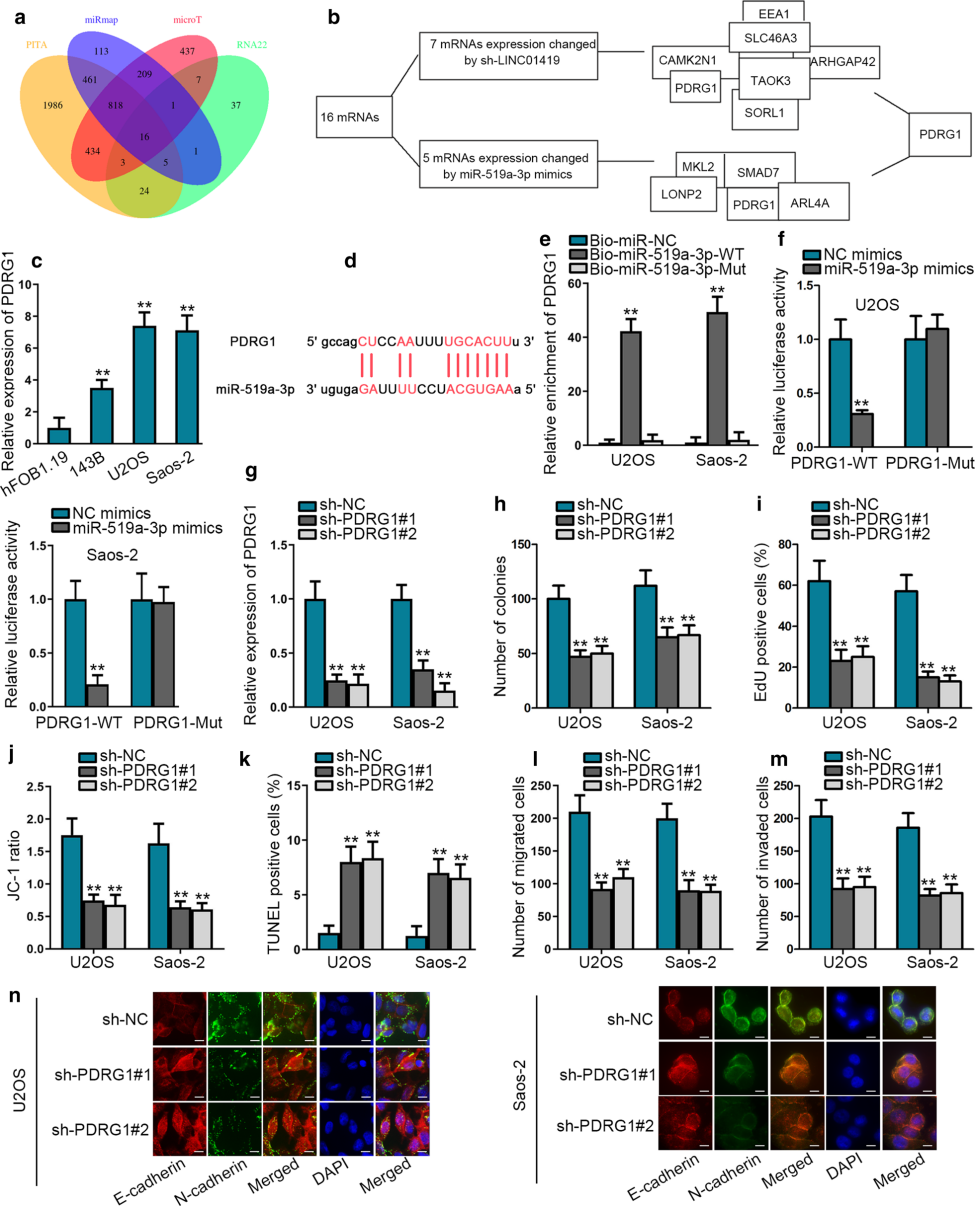
PDRG1 is targeted by miR-519a-3p and promotes OS cell progression. **a** Venn diagram was generated to show the potential mRNAs targeted by miR-519a-3p. **b** The expressions of mRNAs regulated by LINC01419 or miR-519a-3p were shown by qRT-PCR. **c** qRT-PCR analysis was conducted to estimate the expression of PDRG1 in OS cells. **d** Binding site between PDRG1 and miR-519a-3p was presented. **e**–**f**. RNA pull down assay and luciferase reporter assay assessed the combination of PDRG1 with miR-519a-3p. **g** Inhibition efficiency of PDRG1 was detected by qRT-PCR. **h**–**i**. Effect of PDRG1 silencing on U2OS and Saos-2 cell proliferation was evaluated by colony formation and EdU assays. **j**–**k**. The apoptosis of U2OS and Saos-2 cells was examined by JC-1 and TUNEL assays with or without PDRG1 silence. **l**–**m**. The migration and invasion in indicated cells were tested by transwell assay. **n**. IF assay (scale bar = 50 μm) was performed to investigate EMT process in U2OS and Saos-2 cells upon PDRG1 knockdown. ^**^P < 0.01

We further investigated the target genes of miR-519a-3p in OS. Combining with four online tools (PITA, miRmap, microT and RNA22), 16 mRNAs were predicted as the targets of miR-519a-3p (Fig. 3a). Subsequently, among the 16 mRNAs, PDRG1 was screened out since it was the only one that could be regulated by both LINC01419 and miR-519a-3p (Fig. 3b). Hence, we hypothesized that PDRG1 was the downstream molecule of LINC01419/miR-519a-3p axis in OS. Later, we found that PDRG1 was highly expressed in OS cells (Fig. 3c). Furthermore, the binding sequences of miR-519a-3p on PDRG1 3′ UTR was presented in Fig. 3d. Results of RNA pull down assay indicated that PDRG1 was enriched in Bio-miR-519a-3p-WT group rather than Bio-miR-519a-3p-Mut group (Fig. 3e). Luciferase reporter assay further confirmed that upregulation of miR-519a-3p could lessen the luciferase activity of PDRG1-WT but not that of PDRG1-Mut (Fig. 3f). Then, we knocked down PDRG1 expression in OS cells to probe the function of PDRG1 in OS (Fig. 3G). It was discovered that OS cell proliferation was overtly repressed by PDRG1 deficiency (Fig. 3h, i). Besides, the apoptosis of OS cells was accelerated in response to PDRG1 inhibition (Fig. 3j, k). Moreover, we found that silencing PDRG1 restrained the motility of OS cells (Fig. 3l, m). Additionally, EMT process was inhibited in OS cells by PDRG1 interfering (Fig. 3n). To be concluded, PDRG1 was targeted by LINC01419/miR-519a-3p signaling and played an oncogenic role in OS.

### LINC01419 facilitates OS development through targeting miR-519a-3p/PDRG1 axis

**Fig. 4:**
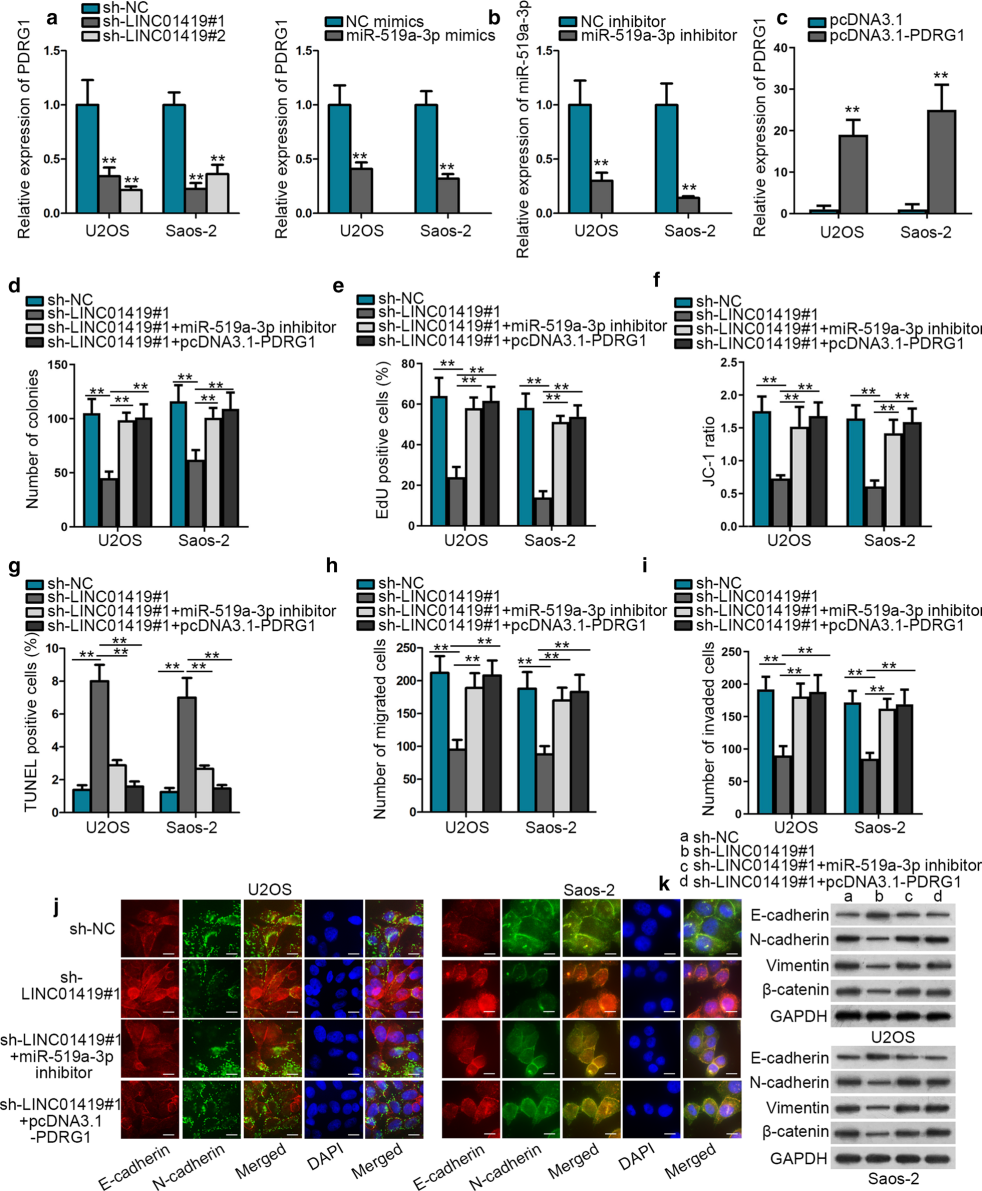
LINC01419 contributes to malignant phenotypes of OS cells through targeting miR-519a-3p/PDRG1 axis. **a** Effect of LINC01419 depletion or miR-519a-3p upregulation on PDRG1 expression was estimated by qRT-PCR. **b**–**c** qRT-PCR analysis was used to determine the transfection efficiency of miR-519a-3p inhibitor or pcDNA3.1-PDRG1. **d**–**e**. Cell proliferation in each group was evaluated by colony formation and EdU assays. **f**–**g**. JC-1 and TUNEL assays were carried out to assess cell apoptosis in each group. **h**–**i**. The motility of cells under indicated transfections was detected by transwell assay. **j**–**k**. IF assay (scale bar = 50 μm) and western blot analysis were employed to test changes on EMT process in each group. ^**^P < 0.01

Finally, we tested the function of LINC01419/miR-519a-3p/PDRG1 axis in OS. At first, we discovered that PDRG1 expression was reduced when LINC01419 was inhibited or miR-519a-3p was overexpressed (Fig. 4a), which confirmed LINC01419/miR-519a-3p/PDRG1 axis in OS. Therefore, restoration experiments were designed and carried out. Prior to that, inhibition efficiency of miR-519a-3p and overexpression efficiency of PDRG1 were validated via qRT-PCR assay (Fig. 4b, c). Based on the results from colony formation and EdU assays, the decreased proliferation caused by LINC01419 inhibition was countervailed by inhibited miR-519a-3p or overexpressed PDRG1 (Fig. 4d, e). On the contrary, JC-1 and TUNEL assays displayed that LINC01419 suppression-augmented apoptosis was offset by miR-519a-3p inhibition or PDRG1 upregulation (Fig. 4f, g). Moreover, miR-519a-3p inhibition or PDRG1 overexpression rescued the restrained motility of LINC01419-depleted OS cells (Fig. 4h, i). Lastly, IF and western blot assays proved that suppressed miR-519a-3p or overexpressed PDRG1 abolished the inhibitory effect of LINC01419 depletion on EMT process (Fig. 4j, k). In conclusion, LINC01419/miR-519a-3p/PDRG1 axis exacerbated cell development in OS.

## Discussion

OS is a musculoskeletal tumor originating in the interstitial cell with a high risk of death [23]. Emerging studies indicated that lncRNAs are closely associated with biological progression of various cancers [24]. It has been reported that lncRNAs with aberrant expression can serve as tumor promoter or suppressor in multiple cancers, including OS [25, 26]. As an example, lncRNA FER1L4 was under-expressed in OS and repressed OS cell proliferation, migration and invasion by miR-18a-5p/PTEN axis [27]. LncRNA B4GALT1-AS1 was up-regulated and contributed to cell migration and stemness in OS by stabilizing YAP mRNA [28]. Similarly, here we found that LINC01419 level was remarkably enhanced in OS tissues and cells. Besides, knockdown of LINC01419 impaired OS cell proliferation, migration, invasion and EMT process while boosted cell apoptosis. Furthermore, LINC01419 silencing inhibited OS tumor growth. These results revealed the oncogenic property of LINC01419 in OS, consistent with its role reported in lung adenocarcinoma, esophageal squamous cell carcinoma and gastric cancer [17–19].

MiRNAs are identified as another subtype of ncRNAs with 20–24 nucleotides in length and play crucial roles in cancer malignant process [29, 30]. Extensive evidence has proved that lncRNAs could serve as a ceRNA of certain miRNAs to block the silence of miRNA downstream genes [31, 32]. The regulatory mechanism between miRNAs and lncRNAs has been widely reported in OS. As illustrated, lncRNA FBXL19-AS1 facilitated cell proliferation and invasion in OS through targeting miR-346 [33]. LncRNA NEAT1 hastened EMT process and predicted poor prognosis by sponging miR-186-5p in OS [34]. The present study indicated that LINC01419 was a cytoplasmic lncRNA that could interact with miR-519a-3p in OS. Importantly, miR-519a-3p was proved to possess a low expression in OS cells. Above data unveiled that LINC01419 exerted a sponging function in OS.

P53 and DNA damage regulated 1 (PDRG1) has been uncovered to accelerate the progression of lung cancer [35]. In addition, PDRG1 functioned as an proliferation-facilitator in gastric cancer [36]. This study uncovered the high level and tumor-facilitating role of PDRG1 in OS cells. Besides, we recognized PDRG1 as the target downstream of LINC01419/miR-519a-3p axis in OS. Restoration assays further confirmed that LINC01419 contributed to OS cell progression via miR-519a-3p/PDRG1 pathway.

## Conclusion

In conclusion, our research was the first to validate the biological function and molecular mechanism of LINC01419 in OS, and confirmed the carcinogenic function of LINC01419/miR-519a-3p/PDRG1 axis in OS development. This finding might provide a theoretic basis for the investigation of OS treatment. However, our study was lack of the exploration on the upstream mechanism of LINC01419 which we would explore in the future.

## Supplementary information

**Additional file 1: Figure S1.** A. LINC01419 expression in 50 pairs of OS tissues and adjacent non-tumor tissues. B. Representative images of tumors excised from sh-LINC01419 and sh-NC group. C-D. Tumor volume and weight were measured in sh-LINC01419 and sh-NC group. E. LINC01419 expression was detected in tumors from sh-LINC01419 and sh-NC group. F. The levels of Ki-67 and PCNA were determined by IHC assay (scale bar = 100 μm). ^**^P < 0.01.

## Abbreviations

OS: Osteosarcoma
lncRNAs: Long noncoding RNAs
LINC01419: Long intergenic non-protein coding RNA 1419
EMT: Epithelial–mesenchymal transition
qRT-PCR: Quantitative real-time polymerase chain reaction
IF: Immunofluorescence
ceRNA: Competing endogenous RNA
miRNAs: MicroRNAs
DMEM: Dulbecco’s Modified Eagle’s Medium
FBS: Fetal bovine serum
GAPDH: Glyceraldehyde-3-phosphate dehydrogenase
shRNA: Short hairpin RNA
EdU: 5-ethynyl-2′-deoxyuridine
PBS: Phosphate buffer saline
SDS-PAGE: Sodium dodecyl sulfate–polyacrylamide gel electrophoresis
PVDF: Polyvinylidene fluoride
IHC: Immunohistochemistry
FISH: Fluorescent in situ hybridization
WT: Wild type
Mut: Mutant type
SD: Standard deviation
ANOVA: One-way analysis of variance
PDRG1: p53 and DNA damage regulated 1
