# Supplementary figures and images for: Long non-coding RNA LINC01419 mediates miR-519a-3p/PDRG1 axis to promote cell progression in osteosarcoma
Source: Cancer Cell Int. 2020 May 5;20:147. doi: 10.1186/s12935-020-01203-0 (PMC7201774; doi:10.1186/s12935-020-01203-0)

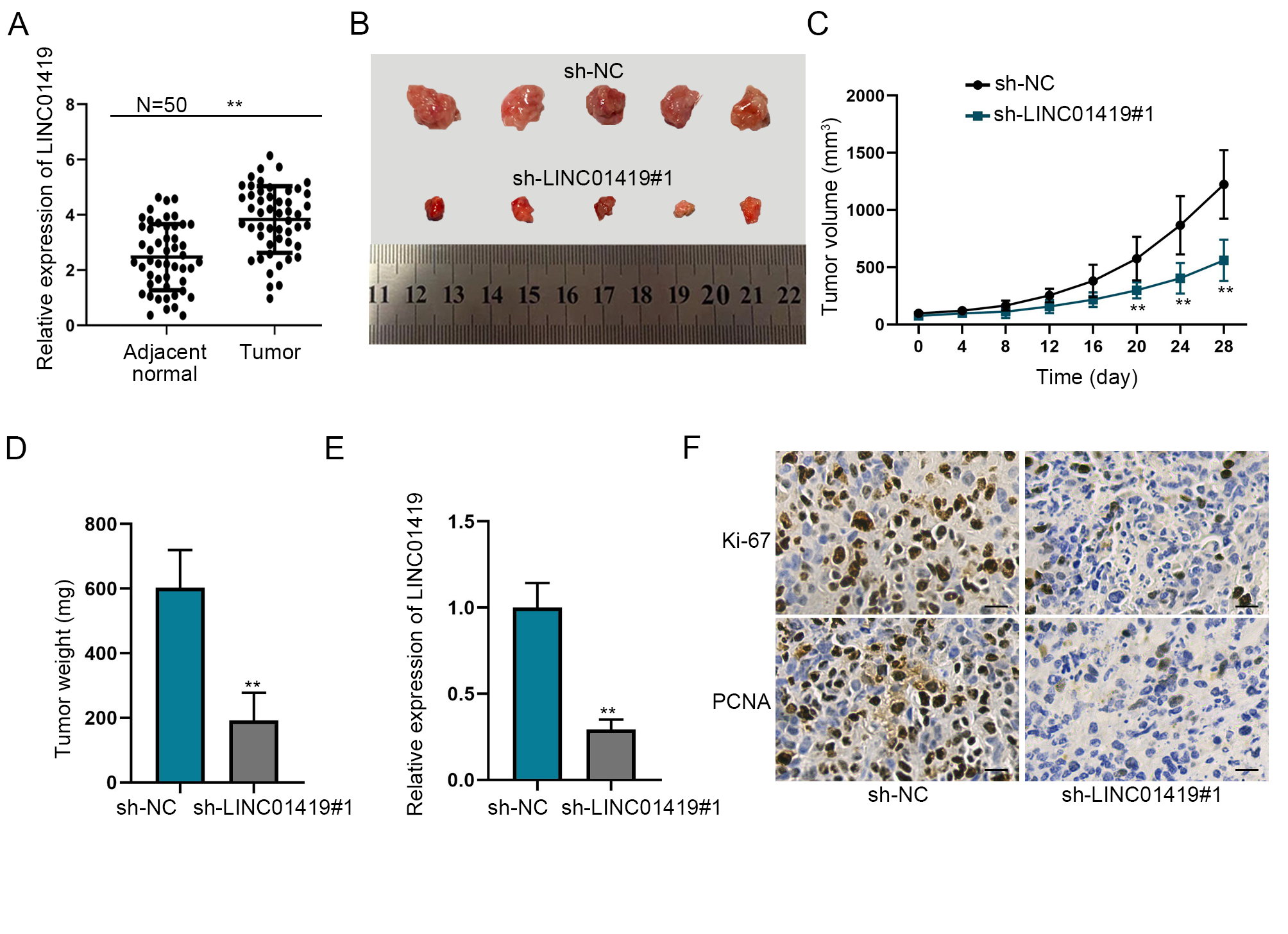

Supplement: Supplementary file 1 — Additional file 1: Figure S1. A. LINC01419 expression in 50 pairs of OS tissues and adjacent non-tumor tissues. B. Representative images of tumors excised from sh-LINC01419 and sh-NC group. C-D. Tumor volume and weight were measured in sh-LINC01419 and sh-NC group. E. LINC01419 expression was detected in tumors from sh-LINC01419 and sh-NC group. F. The levels of Ki-67 and PCNA were determined by IHC assay (scale bar = 100 μm). **P < 0.01. [file 12935_2020_1203_MOESM1_ESM.tif]
